# Whole-Genome Sequence of Beauveria bassiana JEF-350, a Strain with High Insecticidal Activity against Melon Thrips (Thrips palmi)

**DOI:** 10.1128/mra.00470-22

**Published:** 2022-08-08

**Authors:** Jong-Cheol Kim, So Eun Park, Se Jin Lee, Jae Su Kim

**Affiliations:** a Department of Agricultural Biology, Jeonbuk National University, Jeonju, South Korea; b Department of Agricultural Life Science, Sunchon National University, Suncheon, South Korea; c Department of Agricultural Convergence Technology, Jeonbuk National University, Jeonju, South Korea; Vanderbilt University

## Abstract

The entomopathogenic fungus Beauveria bassiana JEF-350 was isolated from forest soil in South Korea. Here, we report the whole-genome sequence of JEF-350, along with the analyzed genetic information, which can be used to study insecticidal mechanisms and fungal diversity.

## ANNOUNCEMENT

Beauveria bassiana (Bals.) Vuill (Ascomycota: Hypocreales) is an arthropod pathogen capable of infecting more than 700 species of hosts, including acari and insecta (1–3). Here, in this work, we provide the whole-genome sequencing (WGS) data of B. bassiana JEF-350 (public stock number for patent, KFCC11820P), which has high insecticidal activity against melon thrips (Thrips palmi), which causes damage to various agricultural crops (4).

The strain was isolated using an insect-baiting method (5) from mountain soil (36°00′53.6″N, 126°47′03.8″E; Gunsan, South Korea) in May 2013 and was stored at −80°C in 20% glycerol solution at the Insect Microbiology & Biotechnology Laboratory (Jeonbuk National University, Jeonju, South Korea). Strain JEF-350 was cultured on quarter-strength Sabouraud dextrose agar medium (BD Difco, Sparks, MD, USA) at 27°C for 7 days prior to sequencing.

Genomic DNA was extracted from JEF-350 using a DNeasy UltraClean microbial kit (Qiagen, Hilden, Germany). The genome was sequenced using both the PacBio Sequel II platform (Pacific Biosciences, Menlo Park, CA, USA) with the SMRTbell template prep kit (Pacific Biosciences) and the Illumina NovaSeq platform (San Diego, CA, USA) with a TruSeq Nano library kit (Illumina) according to the manufacturer’s instructions at Macrogen (Seoul, South Korea). The library insert sizes were 20 kb for the PacBio Sequel sequencing and 350 bp for the Illumina sequencing. In total, 498,137 PacBio subreads were obtained (7,896,107,736 bases; mean length, 15,851 bases; *N*_50_, 23,677 bases). A total of 34,655,292 Illumina reads (5,232,949,092 bases; G+C content, 49.61%; Q30, 91.23%) and 22,202,500 filtered Illumina reads (3,352,577,500 bases; G+C content, 48.37%; Q30, 98.19%) were obtained. The reads were filtered using an in-house script (Macrogen) such that <90% of bases had a quality score of Q30 or higher.

*De novo* genome assembly of the sequenced PacBio reads was conducted using HGAP (6). The assembled contigs were error corrected using Pilon v1.21 (7) to obtain high-quality sequences. There were 8 assembled contigs (35,662,634 bases; *N*_50_, 5,649,914 bases; depth, 199 bases; coverage, 221.4×), and 95% (21,095,181 reads) of the Illumina reads were mapped. The average mapping depth and coverage were 85.98% and 94.0×, respectively.

The locations of protein coding genes were predicted using Maker v2.31.8 (8) with the gene prediction models GlimmerHMM v3.0.4 (9), AUGUSTUS v3.2.2 (10), and SNAP (17 May 2012). Functional annotation of the predicted proteins was performed using protein BLAST+ v2.7.1+ (11) by pattern matching with the UniProt v201806 (12), InterPro v69.0 (13), Pfam v31.0 (14), CDD v3.16 (15), TIGRFAM v15.0 (16), and eggNOG v4.5.1 (17) databases. There were 28,999 and 17,771 exons and introns, respectively. A total of 11,225 protein-coding genes were predicted. In all, 1,593 genes were annotated for transport and metabolism related to amino acids, nucleotides, carbohydrates, coenzymes, lipids, and inorganic ions in the eggNOG classification.

Orthologous analysis of JEF-350 was performed using our previously sequenced isolates, *B.* bassiana ERL836 (GenBank accession number PPTI00000000) and JEF-007 (MRVG00000000) using OrthoMCL v2.0.9 (18). A total of 8,477 genes were shared between the three isolates. Additionally, the numbers of shared genes with JEF-350 were 899 (ERL836) and 391 (JEF-007), and a total of 44 independent genes of JEF-350 were identified. For all software, default parameters were used.

### Data availability.

The whole-genome shotgun sequencing project for B. bassiana JEF-350 has been deposited at DDBJ/ENA/GenBank under BioProject accession number PRJNA616193 and accession number JAAZTN000000000.1. The version described in this paper is JAAZTN000000000.1. The assembled genome sequences are provided under the GenBank accession numbers JAAZTN010000001 to JAAZTN010000008. The Sequence Read Archive (SRA) accession numbers of the raw reads are SRR19115129 and SRR19115130.
